# Supplementary material for: Structural insights into photoactivation of plant Cryptochrome-2
Source: Commun Biol. 2021 Jan 4;4:28. doi: 10.1038/s42003-020-01531-x (PMC7782693; doi:10.1038/s42003-020-01531-x)
Supplement: Supplementary file 2 — Supplementary Information [file 42003_2020_1531_MOESM2_ESM.pdf]

Structural Insights into Photoactivation of Plant Cryptochrome-2

Malathy Palayam[1 †], Jagadeesan Ganapathy[1†], Angelica M. Guercio[1†], Lior Tal[1], Samuel L. Deck[1], and Nitzan Shabek[1*]

Supplementary Information

Supplementary Figure 1: Multiple sequence alignment and conservation analysis of selected cryptochromes and photolyase ortholog.

Supplementary Figure 2: AtCRY2-PHR protein characterization.

Supplementary Figure 3: Structural analysis of CRYs.

Supplementary Figure 4: Characterization of CRYs tryptophan triad and FAD pocket.

Supplementary Table 1: Detailed summary of PHR photo-induced conformational changes and related

Supplementary References

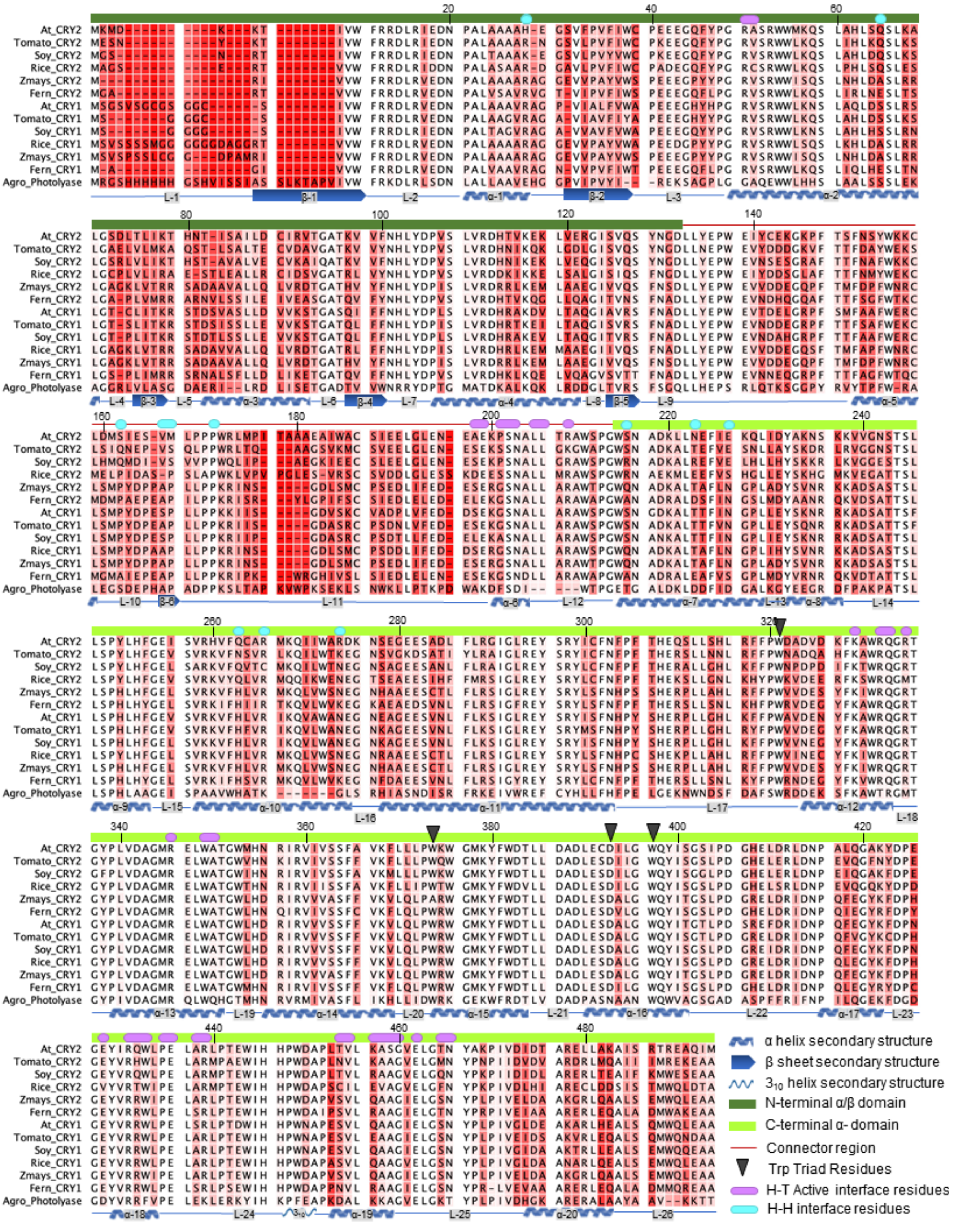
α helix secondary structure
β sheet secondary structure
3₁₀ helix secondary structure
N-terminal α/β domain
C-terminal α- domain
Connector region
Trp Triad Residues
H-T Active interface residues
H-H interface residues
At_CRY2
Tomato_CRY2
Soy_CRY2
Rice_CRY2
Zmays_CRY2
Fern_CRY2
At_CRY1
Tomato_CRY1
Soy_CRY1
Rice_CRY1
Zmays_CRY1
Fern_CRY1
Agro_Photolyase

Supplementary Figure 1. Multiple sequence alignment and conservation analysis of selected cryptochromes and photolyase ortholog. Amino acid alignment of 12 plant cryptochrome proteins with photolyase used as outgroup for comparison. Intensity of red behind residues shows degree of divergence. Numbers on residues refer to position in *At*CRY2-PHR sequence. Key *At*CRY2-PHR subdomains N-terminal α/β domain (dark green), C-terminal α- domain (bright green), and Linker Region (red line) are indicated above alignment. Secondary structure of *At*CRY2-PHR sequence is shown below sequence in blue as alpha helices (α1- α20), beta sheets (β1- β6), 3_{10} helices (3_{10}) and non-secondary structure-containing loops (L1-L26). Black arrow indicates residues involved in Trp triad. Magenta ovals indicate residues involved in formation of H-T interface and Cyan ovals indicate residues involved in formation of H-H interface. In naming scheme At represents *Arabidopsis thaliana*, Tomato *Solanum lycopersicum*, Soy *Glycine max*, Rice *Oryza sativa*, Fern *Asplenium yunnanense*, Zmays *Zea mays*, and Agro *Agrobacterium tumafaciens*.

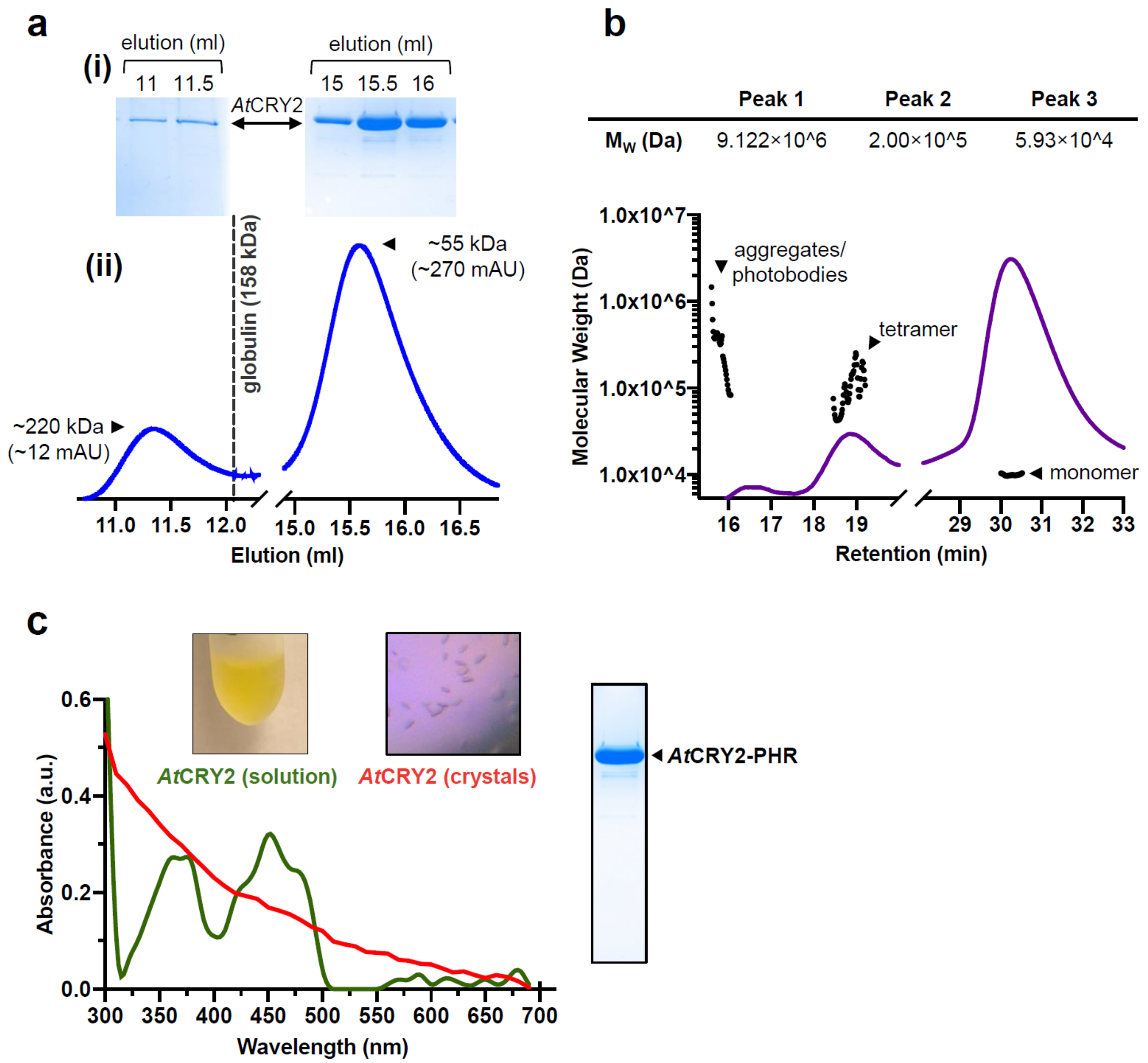

Supplementary Figure 2. AtCRY2-PHR protein characterization. (a) Size exclusion analysis of purified *At*CRY2 PHR domain. MW were estimated by biomolecule standard markers. Elution fractions were resolved by SDS-PAGE and Coomassie staining *(i)* and UV absorption spectra *(ii)*. (**b)** SEC-MALS analysis of *At*CRY2-PHR domain. (**c)** Absorption spectra of *At*CRY2-PHR proteins in solution and in crystals was measured at the range of 300 nm to 700 nm wavelength. Sample of the purified *At*CRY2-PHR used for crystallization trials was resolved by SDS-PAGE and visualized via Coomassie staining.

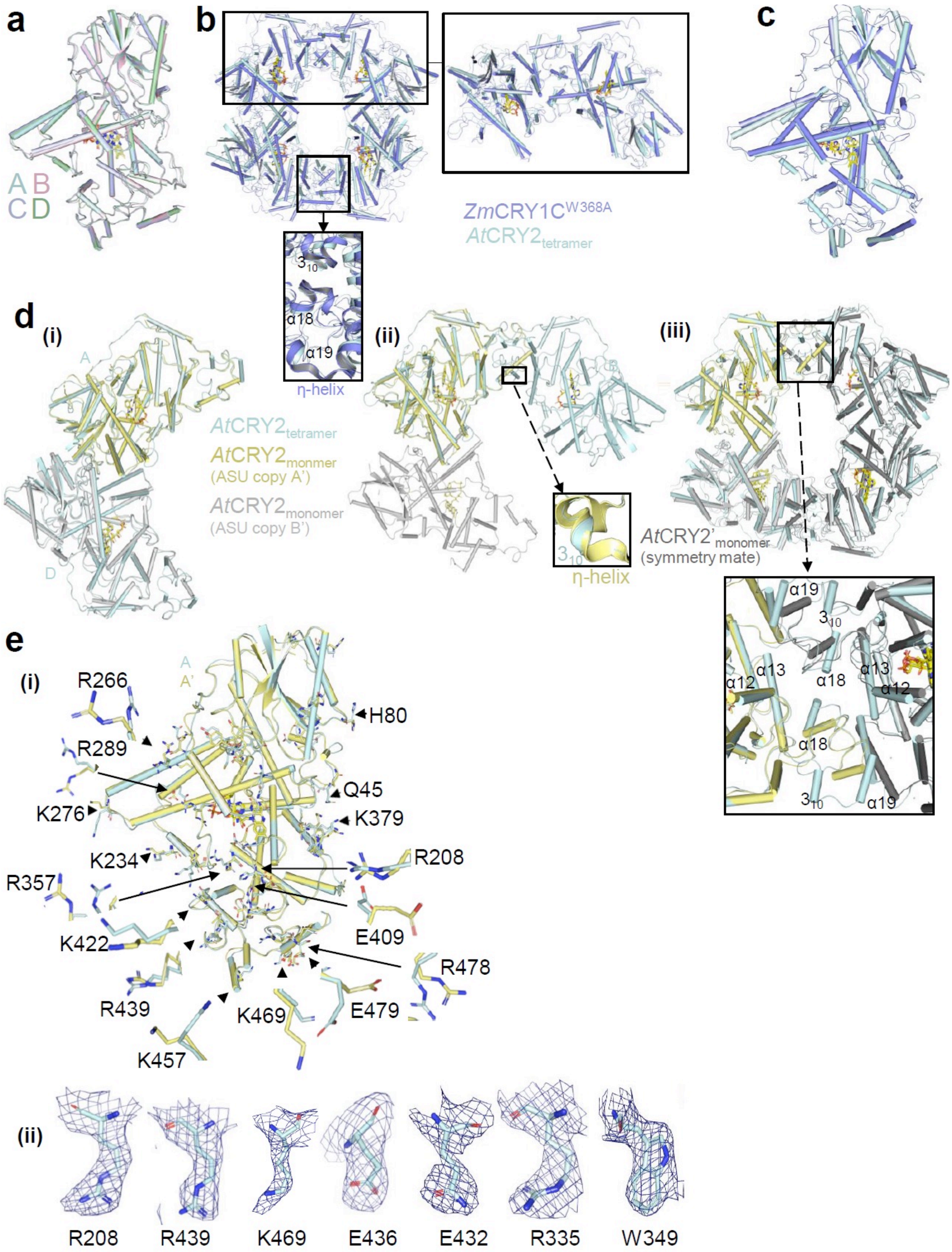
a
A B
C D
b
ZmCRY1C W368A
AtCRY2 tetramer
3 10
α18
α19
η-helix
c
d
(i)
A
D
AtCRY2 tetramer
AtCRY2 monmer
(ASU copy A')
AtCRY2 monomer
(ASU copy B')
(ii)
3 10
η-helix
AtCRY2' monomer
(symmetry mate)
(iii)
α19
3 10
α13
α12
α18
α13
α12
α18
3 10
α19
e
(i)
A
A'
R266
H80
R289
Q45
K276
K379
K234
R208
R357
E409
K422
R478
R439
K469
E479
K457
(ii)
R208
R439
K469
E436
E432
R335
W349

Supplementary Figure 3. Structural analysis of CRYs. (a) Superposition of monomers A (cyan), B (pink), C (purple), D (green) of *At*CRY2-PHR$_{tetramer}$ determined in this study. The r.m.s deviation is calculated for the superposition between monomers A and D (0.59 Å), monomer B and C (0.59 Å) and monomer B and D (0.62Å) chains respectively. **(b-c)** Superposition of *At*CRY2-PHR$_{tetramer}$ (cyan) with mutant *Zm*CRY1C mutant (blue, PDB:6LZ3) resulting in a r.m.s deviation of 1.0Å. Top view of tetramers superposition with close up view on oligomeric interface *(b)* and side view of monomers *(c)*. **(d)** Dimers superposition of *At*CRY2-PHR$_{tetramer}$ (cyan, monomer A and monomer D) with the two copies of *At*CRY2-PHR$_{monomer,}$ (copy A' and copy B' in the Asymmetrical Unit, ASU, shown in yellow and grey respectively, PDB: 6K8I). The two distinct rearrangements resulting in r.m.s.d of 0.88 Å for all the Cα atoms for the alignment in ***(i)***, and r.m.s.d of 0.86 Å for the alignment in ***(ii)***. **(iii)** Superposition of *At*CRY2$_{tetramer}$ (cyan) with the *At*CRY2$_{monomer}$ (yellow, PDB: 6K8I) and its symmetrical mate generated from the crystal structure (crystallographic two-fold symmetry in $C222_1$ space group, *At*CRY2'$_{monomer}$ grey). **(e)** Superposition of *At*CRY2-PHR$_{tetramer}$ (monomer A, cyan) and CRY2-PHR$_{monomer}$ (copy A', yellow, PDB: 6K8I). Side chains with distinct structural deviation are labeled and shown as sticks, close ups of key residues are shown with black arrows ***(i)***. ***(ii)*** Electron density maps of represented rotamers are shown as 2Fo-Fc mesh (σ =1.2).

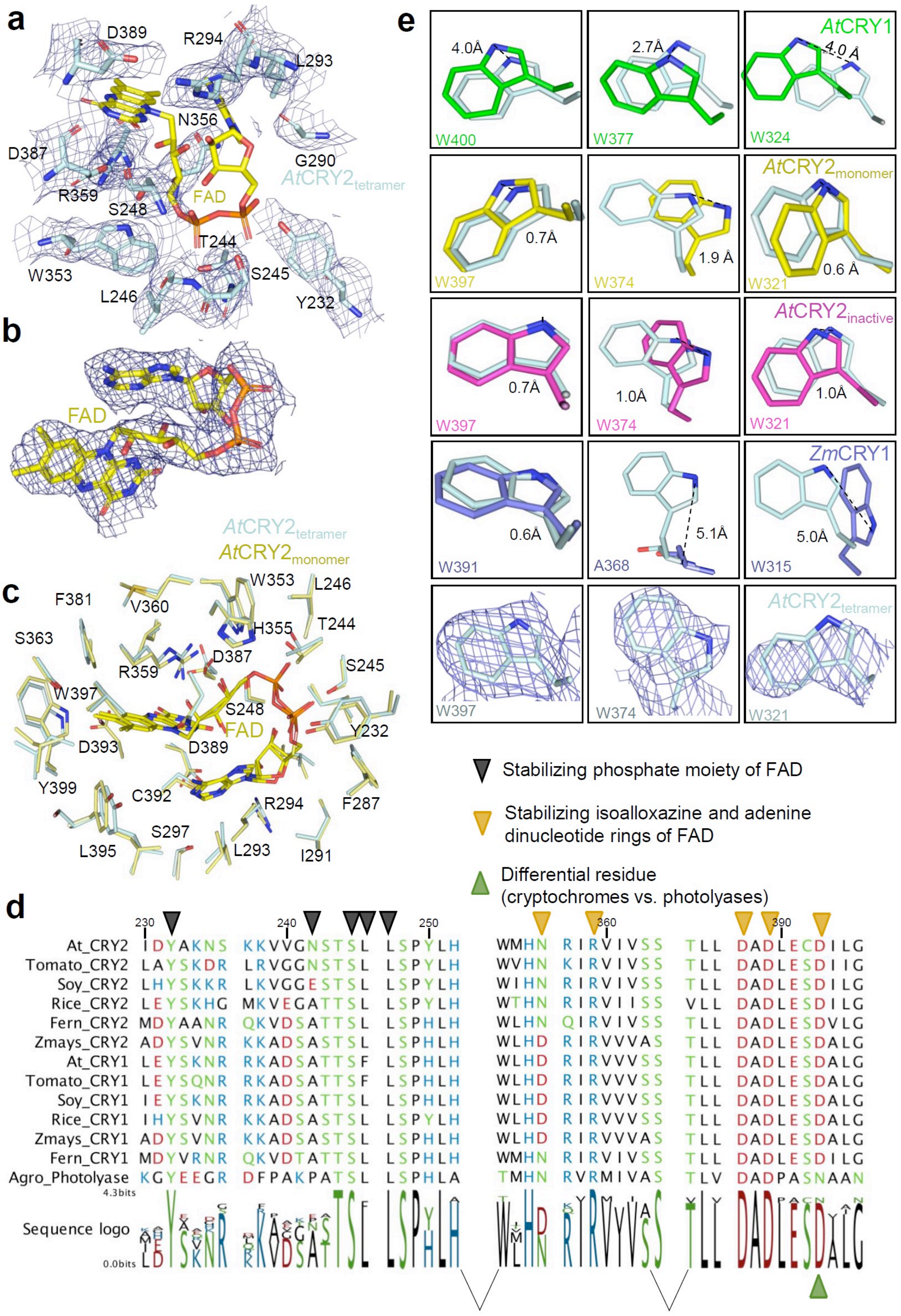
a
D389
R294
L293
N356
D387
G290
AtCRY2tetramer
R359
S248
FAD
T244
W353
S245
L246
Y232
b
FAD
AtCRY2tetramer
AtCRY2monomer
c
F381
V360
W353
L246
T244
S363
H355
D387
R359
S245
W397
S248
FAD
Y232
D393
D389
Y399
C392
R294
F287
S297
L395
L293
I291
d
230
240
250
360
390
At_CRY2 IDYAKNS KKVVGNSTSL LSPYLH WMHN RIRVIVSS TLL DADLECDILG
Tomato_CRY2 LAYSKDR LRVGGNSTSL LSPYLH WVHN KIRVIVSS TLL DADLESDIIG
Soy_CRY2 LHYSKKR LKVGGESTSL LSPYLH WIHN RIRVIVSS TLL DADLESDILG
Rice_CRY2 LEYSKHG MKVEGATTSL LSPYLH WTHN RIRVIISS VLL DADLESDILG
Fern_CRY2 MDYAANR QKVDSATTSL LSPHLH WLHN QIRVIVSS TLL DADLESDVLG
Zmays_CRY2 ADYSVNR KKADSASTSL LSPHLH WLHD RIRVVVAS TLL DADLESDALG
At_CRY1 LEYSKNR RKADSATTSF LSPHLH WLHD RIRVVVSS TLL DADLESDALG
Tomato_CRY1 LEYSQNR RKADSATTSF LSPHLH WLHD RIRVVVSS TLL DADLESDALG
Soy_CRY1 IEYSKNR RKADSATTSL LSPHLH WLHD RIRVVVSS TLL DADLESDALG
Rice_CRY1 IHYSVNR KKADSASTSL LSPYLH WLHD RIRVVVSS TLL DADLESDALG
Zmays_CRY1 ADYSVNR KKADSASTSL LSPHLH WLHD RIRVVVAS TLL DADLESDALG
Fern_CRY1 MDYVRNR QKVDTATTSL LSPHLH WMHN RIRVIVSS TLL DADLESDILG
Agro_Photolyase KGYEEGR DFPAKPATSL LSPHLA TMHN RVRMIVAS TLV DADPASNAAN
4.3bits
Sequence logo
0.0bits
e
4.0Å
2.7Å
AtCRY1
4.0 Å
W400
W377
W324
AtCRY2monomer
0.7Å
1.9 Å
0.6 Å
W397
W374
W321
AtCRY2inactive
0.7Å
1.0Å
1.0Å
W397
W374
W321
ZmCRY1
0.6Å
5.1Å
5.0Å
W391
A368
W315
AtCRY2tetramer
W397
W374
W321
Stabilizing phosphate moiety of FAD
Stabilizing isoalloxazine and adenine dinucleotide rings of FAD
Differential residue (cryptochromes vs. photolyases)

Supplementary Figure 4. Characterization of CRYs tryptophan triad and FAD pocket. (a) 2Fo-Fc electron density map (1σ cut-off) (mesh light blue) of FAD (yellow) binding pocket within *At*CRY2-PHR$_{tetramer}$ (cyan) with the direct interacting amino acids residues shown as sticks. (**b**) 2Fo-Fc electron density map (1σ cut-off) (mesh light blue) of FAD (yellow). (**c**) Close up view on the superposition of *At*CRY2-PHR$_{tetramer}$ with *At*CRY2-PHR$_{monomer}$ (PDB: 6K8I) within the FAD binding pocket. r.m.s.d of 0.4 Å. (**d**) Sequence alignment and conservation of cryptochrome FAD binding pocket across plant species. Colors indicate amino acid polarity. **(e)** Structural analysis of *At*CRY2-PHR$_{tetramer}$ (light blue) tryptophan triad superposition with *At*CRY1 (green, PDB: 1U3C), *At*CRY2-PHR$_{monomer}$ (yellow, PDB: 6K8I), *At*CRY2-PHR$_{inactive}$ (Magenta, PDB:6K8K), *Zm*CRY1C mutant (Blue, PDB:6LZ3).

Supplementary Table 1. Detailed summary of PHR photo-induced conformational changes

Major structural changes		Residue positions	Proposed Function	References
Presence of a short helix		189-192 (*At*CRY2-PHR$_{inactive}$)*<br>181-184 (*At*CRY2-PHR$_{inactive}$)	Photo-inactivation	This study and [1-3]
		189-192 (*At*CRY2-PHR$_{monomer}$)**		
		187-190 (*At*CRY1-PHR)		
		144-146 (*At*CRY1-PHR)		
Absence of a short helix		189-192 (*At*CRY2-PHR$_{tetramer}$)<br>181-184 (*At*CRY2-PHR$_{tetramer}$)	Photo-activation and oligomerization	This study and[1-3]
Presence of 3_{10} helix		448-450 (*At*CRY2-PHR$_{tetramer}$)	Photo-activation and oligomerization	This study
α19 helix	Change of 0.32Å deviation	453-459 (*At*CRY2-PHR$_{inactive}$)	Participate in oligomeric interface	This study and[1-3]
	Change of 0.41Å deviation	453-459 (*At*CRY2-PHR$_{monomer}$)		
	Change of 0.39 deviation	455-463 (*At*CRY1-PHR)		
α18 helix	Change of 0.32Å deviation	427-433 (*At*CRY2-PHR$_{inactive}$)	Participate in oligomeric interface	This study and[1-3]
	Change of 0.32Å deviation	427-433 (*At*CRY2-PHR$_{monomer}$)		
	Change of 0.32Å deviation	430-437 (*At*CRY1-PHR)		
α13 helix	Change of 0.20Å deviation	340-350 (*At*CRY2-PHR$_{inactive}$)	Participate in oligomeric interface	This study and[1-3]
	Change of 0.19Å deviation	340-350 (*At*CRY2-PHR$_{monomer}$)		
	Change of 0.21Å deviation	342-354 (*At*CRY1-PHR)		
α12 helix	Change of 0.24Å deviation	326-333 (*At*CRY2-PHR$_{inactive}$)	Participate in oligomeric interface	This study and[1-3]
	Change of 0.20Å deviation	326-333 (*At*CRY2-PHR$_{monomer}$)		
	Change of 0.15Å deviation	328-337 (*At*CRY1-PHR)		
α6 helix	Change of 0.4Å deviation	201-209 (*At*CRY2-PHR$_{inactive}$)	Participate in oligomeric interface	This study and[1-4]
	Change of 1.3Å deviation	201-299 (*At*CRY2-PHR$_{monomer}$)		
	Change of 1.0Å deviation	199-213 (*At*CRY1-PHR)		

* *At*CRY2-PHR$_{inactive}$ represents the molecular structure of CRY2-PHR in complex with BIC2

** *At*CRY2-PHR$_{monomer}$ represents the molecular structure of CRY2-PHR in monomeric form

Supplementary References

1 Ma, L. *et al.* Structural insights into BIC-mediated inactivation of Arabidopsis cryptochrome 2. *Nat Struct Mol Biol* **27**, 472-479, doi:10.1038/s41594-020-0410-z (2020).

2 Brautigam, C. A. *et al.* Structure of the photolyase-like domain of cryptochrome 1 from Arabidopsis thaliana. *Proc Natl Acad Sci U S A* **101**, 12142-12147, doi:10.1073/pnas.0404851101 (2004).

3 Shao, K. *et al.* The oligomeric structures of plant cryptochromes. *Nat Struct Mol Biol* **27**, 480-488, doi:10.1038/s41594-020-0420-x (2020).

4 Liu, Q. *et al.* Photooligomerization Determines Photosensitivity and Photoreactivity of Plant Cryptochromes. *Mol Plant* **13**, 398-413, doi:10.1016/j.molp.2020.01.002 (2020).
