# Supplementary material for: Structural insights into photoactivation of plant Cryptochrome-2
Source: Commun Biol. 2021 Jan 4;4:28. doi: 10.1038/s42003-020-01531-x (PMC7782693; doi:10.1038/s42003-020-01531-x)
Supplement: Supplementary file 3 — Description of Supplementary Files [file 42003_2020_1531_MOESM3_ESM.pdf]

Description of Additional Supplementary Files

File name: Supplementary Movie 1.

Description: Structural movements between active an inactive CRY2 Morph movie of the conformational variation of the interconnecting loop between active and inactive CRY2 (PDBs: 6X24 and 6K8K).

File name: Supplementary Movie 2.

Description: Overall structural variations between active an inactive CRY2 Morph movie showing the overall structural variation between the active (pale cyan) and inactive CRY2 (PDBs: 6X24 and 6K8K) structures. On the left is the overall structural variations based on superposition. On the right, a close-up view on the amino acids involved in electron transport pathway (represented in sticks).
